# Treatment outcomes of Gene Xpert positive tuberculosis patients in KwaMashu Community Health Centre, KwaZulu-Natal, South Africa: A retrospective review

**DOI:** 10.4102/sajid.v36i1.217

**Published:** 2021-04-07

**Authors:** Sarusha Pillay, Nombulelo P. Magula

**Affiliations:** 1 Internal Medicine, Faculty of Health Sciences, University of KwaZulu-Natal Durban, South Africa

**Keywords:** tuberculosis, sputum conversion, unsuccessful treatment outcomes, Gene Xpert, HIV

## Abstract

**Background:**

We sought to investigate the relationship between tuberculosis (TB) treatment outcomes and its predictors in the KwaMashu region in KwaZulu-Natal (KZN). This area is currently a hotbed for TB and human immunodeficiency virus (HIV) co-infection.

**Method:**

A retrospective study design was adopted to characterise adult patients diagnosed with Gene Expert (GXP) positive pulmonary TB from 01 January 2016 to 31 December 2017. Tuberculosis treatment outcomes were assessed after two months and five months according to the standard World Health Organization (WHO) criteria. Multiple logistic regression analysis was used to calculate the odds ratio (OR) of the possible determinants associated with unsuccessful treatment outcomes.

**Results:**

Amongst the 596 patients diagnosed, 57.4% (95% confidence interval [CI]: 53.3–61.4; 342 of 596) had successful treatment outcomes. Of these reported cases, 88.89% (85.1–92.0; 304 of 342) were cured. For the unsuccessful treatment outcomes, 52.4% (46.0–58.6; 133 of 254) patients were lost to follow-up, 20.9% (16.0–26.4; 53 of 254) failed treatment, 1.2% (0.2–3.4; 3 of 254) died and 25.6% (20.3–31.4; 65 of 254) of the patients could not be accounted for. Patients with unknown HIV status were more likely to have unsuccessful treatment outcomes (adjusted OR [aOR] = 4.94 [1.83–13.36]). Patients who had sputum conversion at 2 months (aOR = 1.94 [1.27–2.96]) were significantly more likely to exhibit unsuccessful treatment outcomes.

**Conclusion:**

Treatment success rate was 57.4% which was below the target set by the WHO. This underscores the urgent need to strengthen treatment adherence strategies to improve outcomes, especially in high HIV burden settings.

## Introduction

In 2018, an estimated 10 million people globally were diagnosed with tuberculosis (TB) with the burden being heterogeneous amongst countries. Of these patients 1.2 million (11%) were co-infected with human immunodeficiency virus (HIV). The worldwide TB mortality was 1.2 million amongst HIV-negative individuals, with 251 000 deaths being reported in HIV-positive patients.^1^ In a recent review of HIV deaths, TB was found to be prevalent in 37% of the deaths; however, in half of the cases, autopsy results indicated that these cases were not diagnosed by the time of death.^2^ The highest TB burden has previously been reported in men (aged ≥ 15 years) with 57% being diagnosed in 2018. Comparatively women accounted for 32% of the cases whilst children (aged < 15 years) accounted for 11%.^1^ Geographically, 30 high TB burden countries collectively accounted for 87% of the world’s cases in 2018.^3^ South Africa has featured amongst the top two-thirds of the countries with the highest incidence of TB globally, with 781 cases per 100 000 being reported in 2016.^4^

To address this silent burden, considerable efforts have been mounted in the past decade to improve TB treatment outcomes and cure rates in South Africa. However, South Africa’s national treatment’s success rate of 76% is still below the expected World Health Organization (WHO) success rate of 90%. Furthermore, the country did not achieve the highly ambitious millennium development goals (MDGs) target of halving TB prevalence and mortality rate.^5^ Coincidentally, in 2017, South Africa reported an incidence of 322 000 TB cases; of these cases, 193 000 (60%) were HIV positive.

This underscores the need to continually strengthen existing TB control initiatives and address its comorbidities in the province.

There has been a steady decline in the incidence rate of TB in South Africa, with 690 per 100 000 cases reported in 2012 compared with 520 per 100 000 in 2015.^6^ In 2015, KwaZulu-Natal (KZN) had the second highest incidence rate of TB of 685 per 100 000 population. However, the most notable decline in the incidence rate has been in KZN over 5 years with a decrease from 1185 to 685 per 100 000 population between 2011 and 2015, respectively.^7^ In 2017, the incidence cases in KZN were 58 117, with almost half, 23 059, cases reported from eThekwini Municipality.

Despite a notable decline in the TB incidence rate both globally and within South Africa, TB still ranks in the top 10 causes of death nationally and worldwide, especially in people living with HIV.^8^ Between 2014 and 2016, TB was the leading cause of death in South Africa.^9^ Being a curable disease still claims 4400 lives daily. In an attempt to control the global burden of disease in 2000, the United Nations MDGs aimed to ‘Stop the spread and reverse the incidence of the global TB epidemic by 2015’. This goal was achieved between 2000 and 2013, and an estimated 37 million lives were saved.^10^ Between 2000 and 2014, the annual incidence rate of TB fell by 1.5%; the Stop TB Partnership which aimed by 2015 to reduce the TB prevalence and mortality rates by 50% compared with 1990 was not achieved worldwide.^11^

As per the WHO, TB treatment outcomes should be assessed annually at both national and district levels.^11^

Knowledge gaps remain on TB treatment outcomes and how they differ by the socio-economic factors in rural settings in KZN. Using routinely captured demographic and clinical characteristics of TB patients presenting at KwaMashu Community Health Centre we sought to evaluate treatment outcomes in a high-risk population group and identify determinants for unsuccessful treatment. This will assist in achieving the Stop TB 90/90/90 targets and also assist South Africa in achieving a 90% success rate.

## Methods

### Study setting

This was a facility-based retrospective study conducted in KwaMashu Community Health Centre.

KwaMashu Community Health Centre is situated in KwaMashu township ward 45 of the eThekwini district, KZN province, South Africa. The centre offers health services to the community of KwaMashu. It has a total of 25 beds. Currently, the centre serves mainly KwaMashu community and surrounding areas, with a catchment population of approximately 750 000 people. It is also a referral centre for six satellite clinics, 11 mobile clinics and other facilities in Phoenix, Inanda, Ntuzuma, Newlands East and Newlands West, Lindelani, and Parlock. In 2012–2013, the Northern district was considered a ‘hot spot’ for the prevalence of HIV, which was estimated at 39%.^12,13^

### Study design and population

This was a retrospective record review of routinely collected data of patients presenting at the KwaMashu Community Health Centre. Demographic and clinical data were retrieved primarily from individual treatment files. Other data sources included provincial TB registers, patient treatment cards and laboratory registers.

Individual patient notes were reviewed to supplement missing data or resolve discrepancies. The study population composed of adult patients (12 years and above) with a treatment outcome recorded between 01 January 2016 and 31 December 2017.

Tuberculosis treatment outcomes were reported and recorded according to the defined WHO outcome categories.^14^ De-identified patient data, including socio-economic and clinical characteristics were collected on a pretested standardised form and entered in an Excel 2013 (Microsoft Corporation, Redmond, USA) spreadsheet. Two individuals independently cross-checked each entry for logical inconsistencies, invalid codes, omissions and improbable data by tabulating, summarising and plotting variables with missing observations being excluded systematically.

### Key terms in accordance to World Health Organization definitions and reporting framework for tuberculosis

#### Cured

An initially sputum smear-positive patient whose sputum smear or culture is negative at or one month prior to the completion of TB treatment and on at least one previous occasion at least 30 days prior.

#### Treatment completed

A patient whose baseline sputum was positive and who has completed anti-TB treatment without evidence of failure but for whom sputum smear or culture results are not available in the last month of treatment and on at least one previous occasion.

#### Treatment success

The sum of patients who were declared ‘cured’ and those who had ‘completed’ treatment.

#### Treatment failure

A patient whose sputum smear or culture is positive at the fifth month of treatment or later during the course of treatment. Excluded in this definition are patients found to have multi-drug-resistant TB at any point of time during the treatment.

#### Treatment defaulter or lost to follow-up

A patient who has been on treatment for at least four weeks and whose treatment was interrupted for two consecutive months or more.

#### Died

A patient who died because of TB or for any reason during the course of treatment.

#### Transfer out

A patient who started treatment and was transferred to another treatment unit and for whom the treatment outcome is not known at the time of evaluation of treatment results.

*Successful treatment* composed of TB patients who were cured and those who completed their treatment, whereas *unsuccessful treatment* outcomes are TB patients who died, failed treatment, were lost to follow-up or not evaluated.

### Statistical analysis

Data were analysed in STATA 15 (Stata Corp LP, College Station, TX, USA). Summary statistics were presented, with continuous variables presented with their mean and standard deviation (SD), whereas categorical variables summarised as counts and proportions (%). A univariate and multivariate logistic regression models were used to evaluate the relationship between unsuccessful treatment outcome (dependent variable) and the selected demographic and clinical variables. The variables examined were gender, HIV status, age, antiretroviral treatment (ART) uptake, diabetes mellitus and smear microscopy results. To determine the effect of these variables on unsuccessful treatment, unadjusted and adjusted odds ratios (aORs), 95% confidence interval (CI) and *p-*value were used to assess the associative strength for each predictor with a two-sided *p* < 0.05 deemed statistically significant.

### Ethical considerations

Ethical clearance and approval for the study was provided by the Biomedical Research Committee (BREC) in association with the University of KZN (BREC No. BE509/16). Patients’ records were anonymised and de-identified before analysis with strict confidentiality of patient information prioritised.

## Results

### Study population

A total of 596 patients’ records were considered over the study period. The largest proportion of patients reported were males 63.9% (381/596), females were 36.1% (215/596). A total of 360 (60.4%, 95% CI: 56.3–64.4) of the patients examined were HIV positive. Majority of the HIV patients 69.4% (95% CI 64.4–74.2; 250/360) were reported as being on ART. The 30–39 age range had the highest percentage (34.2%) of TB cases, followed by the 20–29 age range with (32.2%), 40–49 age range with 15.9%, 50–59 range with 7.6%, below 20 with 6.0% and above 60 with 4.0% (Table 1).

**TABLE 1:** Demographic characteristics of tuberculosis patients attending KwaMashu Community Health Centre, KwaZulu-Natal, South Africa.

Patient’s characteristics	Variable	Patients	
		*n*	%
Gender	Male	381	63.9
	Female	215	36.1
HIV status	Positive	360	60.4
	Negative	168	28.2
	Unknown	68	11.4
ART (*n* = 360)	Yes	250	69.4
	No	99	27.5
	Unknown	11	3.1
Age (years)	< 20	36	6.0
	20–29	192	32.2
	30–39	204	34.2
	40–49	95	15.9
	50–59	45	7.6
	> 60	24	4.0
Smear microscopy (2 months)	Yes	388	65.1
	No	208	34.9
Smear microscopy (5 months)	Yes	257	43.1
	No	337	56.5
	Unknown	2	0.4

HIV, human immunodeficiency virus; ART, antiretroviral therapy.

### Treatment outcomes

Treatment outcomes of new smear positive pulmonary TB patients as per the standard WHO reporting criteria are shown in Table 2.

**TABLE 2:** Treatment outcomes of new smear positive pulmonary tuberculosis patients in KwaMashu Community Health Centre, KwaZulu-Natal, South Africa, as per the standard criteria (*N* = 596).

Treatment outcomes	Patients	
	*n*	%
**Successful**		
Cure	304	51.01
Treatment completed	38	6.38
**Unsuccessful**	254	42.62
Treatment failure	53	8.89
Treatment defaulter	133	22.32
Died	3	0.50
Unaccountable	65	10.91

**Total**	**342**	**57.38**

Out of the 596 patients included in the study 51.01% (*n* = 304) were cured, 6.38% (*n* = 38) completed their treatment, 8.89% (*n* = 53) had treatment failure at five months, 22.32% (*n* = 133) were lost to follow-up, 0.50% (*n* = 3) died and 10.91% (65/596) of the patients’ treatment outcomes could not be accounted for (Table 3).

**TABLE 3:** Clinical characteristics of tuberculosis patients in KwaMashu Community Health Centre, KwaZulu-Natal, South Africa, stratified by gender.

Patient’s characteristics	Variable	Patients				Total	
		Male		Female		*n*	%
		%	95% CI	%	95% CI		
Age (years)	< 20	36	22.1–53.0	64	47.0–77.9	36	6.0
	20–29	58	51.2–65.1	42	34.9–48.8	192	32.2
	30–39	69	62.4–75.1	31	24.9–37.6	204	34.2
	40–49	72	61.6–79.8	28	20.2–38.4	95	15.9
	50–59	67	51.6–78.9	33	21.0–48.4	45	7.6
	> 60	71	49.7–85.7	29	14.3–50.3	24	4.0
HIV status	Positive	57	52.0–62.3	43	37.7–48.0	360	60.4
	Negative	72	64.7–78.3	28	21.7–35.3	168	28.2
	Unknown	79	68.1–87.5	21	12.5–31.9	68	11.4
ART (HIV +ve)	Yes	56	49.7–62.1	44	37.9–50.2	250	69.4
	No	61	50.6–69.7	39	30.2–49.4	99	27.5
	Unknown	55	25.6–80.7	46	19.3–74.4	11	3.1
Diabetes mellitus	No	64	60.3–68.4	36	31.6–39.7	529	96.5
	Yes	56	32.4–76.5	44	23.5–67.6	18	3.3
	Unknown	0	-	1	-	1	0.2
Smear microscopy result	Negative	61	54.6–67.1	39	32.9–45.4	236	39.6
	Positive	69	62.5–74.0	32	26.0–37.5	251	42.1
	Unknown	60	50.1–68.5	40	31.5–49.9	109	18.3

HIV, human immunodeficiency virus; ART, antiretroviral therapy; CI, confidence interval.

Although not statistically significant, male patients (OR = 1.04 [0.73–1.49]), patients with HIV (OR = 1.25 [0.85–1.82]), and patients between 30 and 39 years (OR = 1.07 [0.50–2.2]7) and patients above 60 years (OR = 1.50 [0.47–4.76]) were more likely to have unsuccessful treatment outcomes. On the contrary, patients with unknown HIV status (OR = 2.60 [1.32–5.14]) and those who had their sputum conversion at 2 months (OR = 1.88 [1.27–2.78]) were more likely to have unsuccessful treatment outcomes.

Multiple logistic regression analysis was used to jointly explore the determinants of unsuccessful treatment outcomes in the study cohort. The independent determinants for unsuccessful treatment were gender, HIV status, ART, age, diabetes and sputum conversion at two months. After adjusting for possible confounding factors, patients with unknown HIV status (aOR = 4.94 [1.83–13.36]) and patients who had their sputum conversion at 2 months (aOR = 1.94 [1.27–2.96]) were significantly (*p* < 0.05) more likely to have unsuccessful treatment outcomes (Table 4).

**TABLE 4:** Determinants of unsuccessful treatment outcome in KwaMashu Community Health Centre, KwaZulu-Natal, South Africa.

Variable	Unadjusted odds ratio			Adjusted odds ratio		
	OR	95% CI	*p*	aOR	95% CI	*p*
**Gender**						
Female	-	-	-	-	-	-
Male	1.040	0.730–1.490	0.814	1.020	0.660–1.570	0.941
**HIV status**						
Negative	-	-	-	-	-	-
Positive	1.250	0.850–1.820	0.257	1.590	0.860–2.970	0.142
Unknown	2.600	1.320–5.140	0.006	4.940	1.830–13.360	0.002
**ART**						
No	-	-	-	-	-	-
Yes	0.840	0.590–1.190	0.333	0.700	0.390–1.230	0.213
Unknown	0.670	0.270–1.700	0.402	0.350	0.080–1.420	0.140
**Age (years)**						
Below 20	-	-	-	-	-	-
20–29	0.980	0.460–2.080	0.952	1.090	0.430–2.770	0.864
30–39	1.070	0.500–2.270	0.862	1.110	0.420–2.930	0.829
40–49	0.940	0.420–2.110	0.880	0.960	0.340–2.720	0.943
50–59	1.000	0.390–2.530	1.000	1.230	0.390–3.930	0.723
Above 60	1.500	0.470–4.760	0.491	2.300	0.460-11.400	0.309
**Diabetes**						
No	-	-	-	-	-	-
Yes	0.990	0.370–2.700	0.996	0.720	0.210–2.460	0.602
**Sputum conversion 2 months**						
No	-	-	-	-	-	-
Yes	1.880	1.270–2.780	0.002	1.940	1.270–2.960	0.002

CI, confidence interval; HIV, human immunodeficiency virus; ART, antiretroviral treatment.

## Discussion

In this retrospective study, we assessed TB treatment outcomes and the plausible determinants of unsuccessful outcomes. The study findings revealed a higher percentage of TB cases amongst the age groups of 20–29 and 30–39. This is consistent with studies done in Mpumalanga South Africa, Northeast and Central Ethiopia.^15,16^ In our study, the disproportionate burden in the age groups 20–29 and 30–39 could be as a result of greater mobility attendant to this demographic group occasioned by social and economic ambitions. A further contributing factor within the South African population could be because of the high prevalence of HIV co-infection amongst this age groups.^17^ However, these findings are in contrast to studies in China, Cambodia and Vietnam where patients older than 55 years had low incidence of TB.^18,19^ Diabetes, concomitant malignancies and other co-morbidities in the elderly have previously been identified as predisposing for the elderly.^20^ Further evidence alludes age as an important factor in determining TB treatment outcomes.^19,21,22,23^ However, in our cohort, no significant relationship was found between all the age groups and unsuccessful treatment outcome.

We observed a higher proportion of TB amongst all cases to be higher amongst males than females.

A finding similar to other international studies conducted in Malaysia (70.24%),^24^ Cambodia, China, Vietnam (69.1%),^18^ Ethiopia (61.3%)^25^ and Pakistan (51.7%)^26^ which have noted a similar trend. This could be attributed to differential susceptibility to TB because of biological orientation, lower female notification rates as a result of socio-economic and cultural barriers in healthcare access and high exposure risk in males because of their high-risk exposure, substance abuse and social engagement.^27^ In addition, young adults (30–39 years) and older adults (above 60 years) represent a vulnerable population cohort with unique challenges with regard to TB treatment.

For the younger population, this might be attributed to poor adherence to treatment and behaviours/lifestyle choices that may make them more vulnerable to unsuccessful outcomes.^28,29^

A large proportion of the patients in this study (60.4%) were HIV positive, consistent with HIV and TB coinfection rates in South Africa.^1^ Cluster of differentiation 4 (CD4) cell counts and viral loads were not known in many patients. Of note, only 69.4% of the HIV-positive patients were on ART. This is of concern as according to the Joint United Nations Programme on HIV/AIDS (UNAIDS) strategy to end HIV as a public health threat by 2030, 90% of all people living with HIV should know their HIV status, 90% of all patients diagnosed with HIV infection should receive sustained ART and 90% of all people receiving ART should have viral suppression by 2020.^30^ With the institution of the ‘test and treat’ campaign in 2016 together with the national TB guidelines, it is of concern that there is a large number of patients who are not on ART.^31,32^

Our study also revealed that 11% of patients were also unaware of their HIV status.

One of the determinants of an unsuccessful treatment outcome in this study was patients who did not know their HIV status. An opt-out approach for HIV testing should become a routine procedure as opposed to an opt in approach. This would result in less patients with HIV/TB co-infection being missed and allow for earlier diagnosis and treatment.^33,34^ Patient diagnosed and initiated on treatment during the advanced stages of HIV could also have affected treatment outcomes.^35^

Previous evidence suggests that patients with higher CD4 cell counts and who are on ART have been shown to have more successful outcomes.^36^ Tuberculosis and HIV treatment in combination has been shown to improve treatment failure rates.^21^ For patients who are HIV and TB coinfected and on ART, the national rates have improved from 28% in 2011 to 89.1% in 2017.^7^ In comparison to the national standards, our study showed a large proportion of HIV positive patients who were not on treatment. Stronger initiatives aimed at HIV testing and commencement of treatment, especially in TB and HIV high-risk areas will need to be used to improve TB treatment outcome rates.

It is important to achieve and sustain acceptable levels of treatment success rates amongst TB patients.^21,37^ The overall treatment success rate based on sputum smear positive TB cases was 57.38%. The cure rate was 51.01%.

The treatment success rate obtained in our study is relatively higher than 40.6% in Uganda.^38^ However, this was still low compared with the WHO successful target rate of 90%.^39^ Countries which have succeeded in making this target are Ethiopia (91.8%)^40^ and China (95.02%, 93.9%).^41,42^ A systemic review and meta-analysis conducted in seven Sub-Saharan Africa countries showed a total TB success rate of 76%.^43^ Two studies in Gauteng Province found success rates of 80%^44^ and 83.48%.^2^ The low TB success rate within our study is an indication of generally underperforming and failing TB programme in resource-constrained areas.

Other contributing factors might be our study’s sample size, the study period, HIV status and healthcare quality.

A possible reason for the higher success rates obtained in the Gauteng studies may be the inclusion of all TB patients regardless of smear status, extrapulmonary TB and children.

Patients who were transferred out were also excluded from the Gauteng study. There was also a large default group in our cohort. A large pill burden associated with HIV and TB coinfection as well as patients starting to feel better after initial treatment commencement may have resulted in patients defaulting treatment. The KwaMashu district remains an impoverished community with a high rate of unemployment. Financial factors in terms of accessing healthcare facilities and attending follow-up visits may have also contributed to patients defaulting treatment.

A deeper understanding of factors associated with unsuccessful TB treatment outcomes can encourage appropriate interventions intended to reduce morbidity and mortality.^42^ In this study, loss to follow-up 22.32% (*n* = 133) composed a major portion of the unsuccessful outcome. Significantly lower rates were noted in a primary healthcare facility in Johannesburg (11%),^44^ Gauteng province (5.4%)^23^ and Ethiopia (8.5%).^15^ Age, gender and not being on ART have shown to increase the risk for lost to follow-up.^23^ Several reasons related to our study participants and the health centre may have contributed to the outcomes reported. It was noted in this study that patients were lost to follow-up because of patient relocation; attempts were made by healthcare workers to trace patients through home visits and telephonic consultations. Social issues including alcohol and drug abuse as well as psychiatric conditions also contributed to patients being lost to follow-up.

Sputum conversion at two months was significantly associated with unsuccessful TB treatment outcomes. These findings are inconsistent with studies widely reported in Western Cape, South Africa,^45^ Pakistan^26^ and India.^46^ A possible reason for this could be that a large proportion of the unsuccessful outcomes in our study were because of patients who were lost to follow-up. Patients who had achieved sputum conversion at two months may have believed that they have been cured and thereafter defaulted further treatment and follow-up. Despite diabetes not showing a statistical significance relationship with treatment outcomes in our study, diabetes is known to reduce a patient’s immune response, thus making them more susceptible in acquiring the infection as well as having poorer outcomes.^47,48^ Future treatment and monitoring programmes and strategies should aim to ensure that patients are followed up for the entire six months and that patients adhere strictly to treatment regimes.

## Study limitations

This was a retrospective study in a rural health community that is bombarded with unmet health needs for HIV-infected patients presenting with possible TB and the inherent limitations of registry-based data. Information on important variables such as patients’ CD4 count, education-level HIV risk factors and hospitalisation history was not identified. Study results should be applied with caution, as we were unable to verify HIV status or validate TB laboratory results. We were also not able to accurately link all our patients to the various outcomes. It is also possible that patients considered in our study might have been lost or misclassified in the present analysis.

## Conclusion

Tuberculosis continues to be the most common presenting illness amongst people living with HIV in rural South Africa. The study findings showed the treatment success rate to be below the WHO target of 90%. Information about determinants of unsuccessful treatment outcomes provides important insight for steering TB and HIV control initiatives to meet the needs of different age and risk groups in rural settings in South Africa. The study serves as a quality improvement indicator that can hopefully be extrapolated onto a bigger scale and promote similar studies to be undertaken in other provinces grappling with improving TB treatment outcomes.
